# Design of dispersant for highly concentrated one-dimensional Nb_2_Se_9_ inorganic molecular chains from bulk crystal

**DOI:** 10.1038/s41598-019-51210-z

**Published:** 2019-10-10

**Authors:** Sudong Chae, Akhtar J. Siddiqa, Seungbae Oh, Bum Jun Kim, Kyung Hwan Choi, Hak Ki Yu, Jae-Young Choi

**Affiliations:** 1 School of Advanced Materials Science & Engineering, Sungkyunkwan University, Suwon, 16419 Korea; 2 SKKU Advanced Institute of Nanotechnology (SAINT), Sungkyunkwan University, Suwon, 16419 Korea; 3 Department of Materials Science and Engineering & Department of Energy Systems Research, Ajou University, Suwon, 16499 Korea

**Keywords:** Synthesis and processing, Synthesis and processing

## Abstract

We determined the optimum dispersant to separate bulk Nb_2_Se_9_ material into 1D chain units. The Nb_2_Se_9_, which had a negative zeta potential (−43.3 mV), showed acidic characteristics and strongly bonded with the amine head of octadecyl amine through a charge transfer (from the amine to Se atoms) reaction. The steric hindrance of the octadecyl tail resulted in excellent dispersion of Nb_2_Se_9_ (down to nanometre-sized mono-chains).

## Introduction

Low-dimensional materials have opened new areas of research, and their advanced applications have attracted the interest of the materials community. In particular, two-dimensional (2D) materials such as graphene, transition metal dichalcogenides (TMDCs), and black phosphorous have been attracting much attention due to their unique physicochemical and electromagnetic properties, and many studies have been conducted on transistors, superconductors and optoelectronic devices^1–5^. Unlike the low-dimensional material obtained by simply reducing the size from the bulk material, the above-mentioned 2D materials are obtained by physically or chemically exfoliating the material having the weak interlayer binding by van der Waals (vdW) force. The resulting 2D materials have unique structural features, such as atomic thicknesses, a lack of dangling bonds on their surfaces, and flexibility.

Recently, 1D materials such as Mo_6_S_3_I_6_^6–9^, and Mo_6_S_4.5_I_4.5_^10,11^, Sb_2_S_3,_ and Sb_2_Se_3_^12–14^, which have similar structural features to 2D materials but different dimensionalities, have been intensively studied. These 1D materials were obtained by exfoliating bulk 1D materials into nanowires or molecular chains, because bulk 1D materials have weak van der Waals (vdW) interactions between unit inorganic chains with strong covalent bonds. Isolated inorganic chains have very interesting structural characteristics for device applications, including diameters less than 1 nm, no dangling bonds on the chain surfaces, high aspect ratios, and flexibility like 2D materials. Owing to these structural features, they have very unique physical and chemical properties, leading to very useful applications, such as molecular connectors, transistors, sensors, photovoltaic devices, and composites^6–8,15–20^. In addition, very recently, Sb_2_S_3_ and Sb_2_Se_3_ were reported to have excellent optoelectronic properties because they have no dangling bonds on their chain surfaces^12–14^.

Recently, the authors successfully prepared a novel 1D bulk crystal, Nb_2_Se_9_. The crystal was synthesised by a chemical reaction between Nb and Se in an evacuated quartz ampoule, could be reproduced in large quantities, and was stable in air^21,22^. These properties are essential for use in subsequent processes and device applications. It is important to isolate inorganic nanoscale chains from this bulk crystal to study the material properties or device applications^23–25^. For this purpose, the authors dispersed Nb_2_Se_9_ crystals in a solvent because the solvent exfoliation method can be used to obtain large sample quantities simply and hence, has been widely used for dispersing CNT bundles^26,27^ and 2D materials^28^. However, it was very difficult to prepare a high-concentration solution by dispersing Nb_2_Se_9_ crystals with only a solvent in our experiment^29^. Furthermore, the surface characteristics of the Nb_2_Se_9_ crystals and the functional groups that can be adsorbed effectively remain unexplored. In this study, we designed the chemical structure of a dispersant for the nano-dispersion of Nb_2_Se_9_ crystals to prepare a highly concentrated solution of Nb_2_Se_9_ nanowires and to verify whether single molecular chains could be obtained from this solvent. This not only provides basic information on the surface of Nb_2_Se_9_, but it can also provide guidelines for further chemical reactions utilizing it.

## Result and Discussion

**Figure 1:**
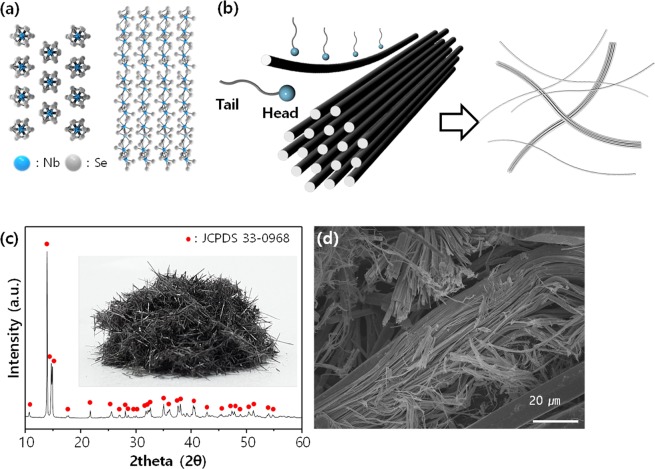
(**a**) Ball-and-stick crystal structure of Nb_2_Se_9_; (**b**) schematic of exfoliation process by dispersant, (**c**) XRD pattern of the Nb_2_Se_9_ crystal (inset: the digital photograph of synthesized Nb_2_Se_9_ crystal), (**d**) SEM image of the Nb_2_Se_9_ crystal.

Nb_2_Se_9_ is composed of chain-shaped molecular units with strong covalent bonds (Nb atoms are decorated by Se atoms) that are assembled in a crystal structure *via* weak vdW attractions between chains (Fig. 1a). During dispersion, mono-chains can be exfoliated from the 3D crystal due to the weak interactions between chains (Fig. 1b). Single crystalline Nb_2_Se_9_ was grown via a chemical reaction between Nb and Se in an evacuated quartz ampoule. When the Nb-Se mixture at 700–800 °C was cooled to room temperature, dark grey needle-shaped bulk crystals were formed, and XRD analysis confirmed that the material had a well-crystallised Nb_2_Se_9_ phase (Fig. 1c). Figure 1d shows the SEM image of the Nb_2_Se_9_ crystals prepared in this study. It was observed that some Nb_2_Se_9_ was naturally exfoliated in the form of a chain. Consequently, the material synthesised in this study could be dispersed as 1D units using a suitable dispersant.

**Figure 2:**
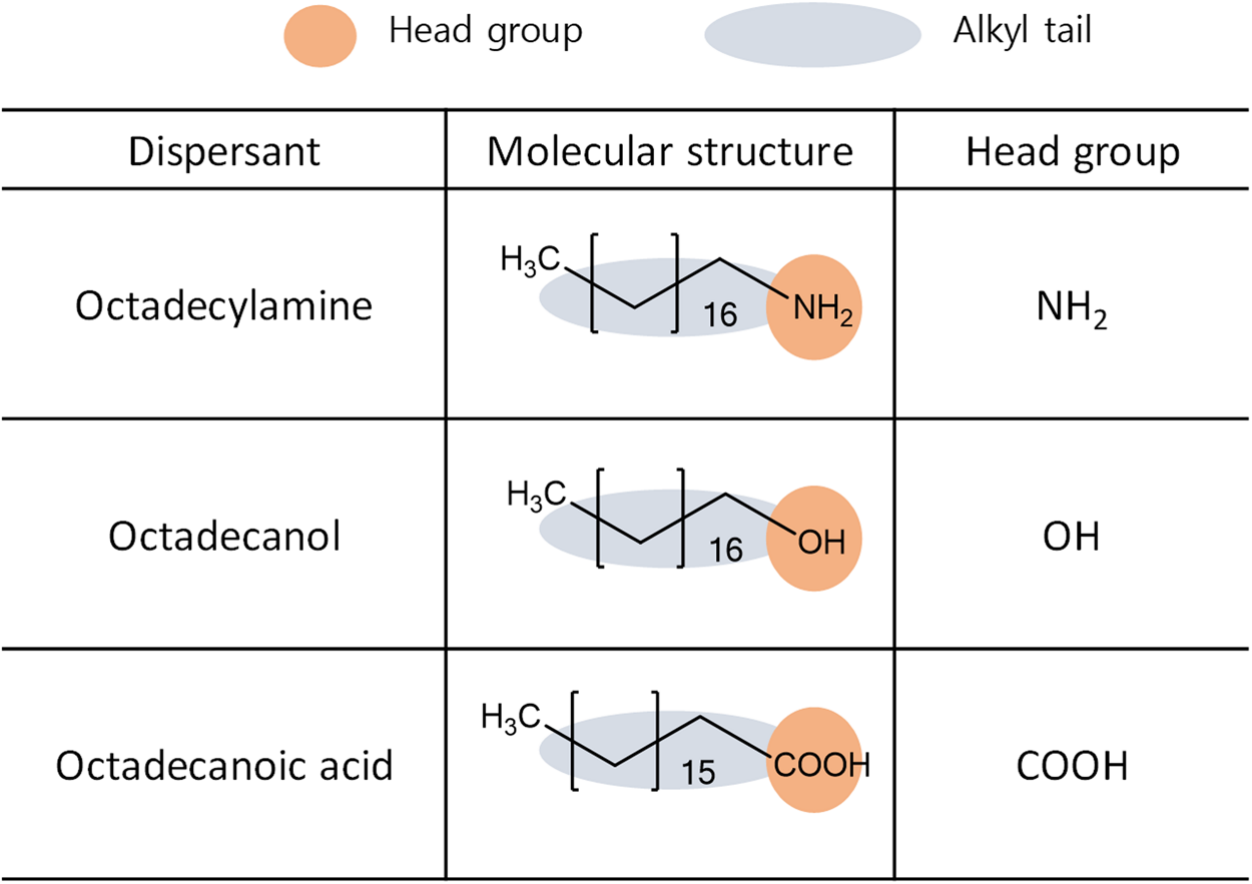
Basic information of designed dispersant.

In general, the structure of a dispersant is composed of head and tail groups. To obtain nanoparticles dispersed at a high concentration using a dispersant, the following two conditions must be satisfied^30^. First, the head groups of the dispersant must be firmly fixed to the surfaces of the nanoparticles. Second, the tail groups of the dispersants should be highly solubilised to strongly repel the nanoparticles through steric hindrance. In this experiment, 2-propanol (IPA) was used as the main solvent of the Nb_2_Se_9_ dispersant because it showed good dispersing performance in a test experiment using only the solvent. Three types of head groups were considered for the Nb_2_Se_9_ dispersion: an acidic carboxylic acid group, a neutral hydroxyl group, and a basic amine group. The octadecyl-alkyl group, which is highly soluble in IPA and has a linear chain structure (for strong steric hindrance), was selected as the tail of the dispersant. Figure 2 shows the structure of the dispersants used in this study.

**Figure 3:**
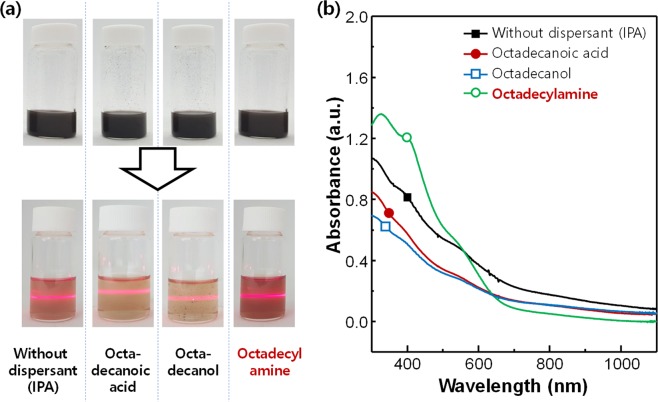
(**a**) Digital photographs of dispersion solutions after ultrasonication (top) and separated supernatants after centrifugation with Tyndall effect (bottom). (**b**) UV-vis-NIR absorption spectra of the dispersion solutions.

Nb_2_Se_9_ crystals in solution were dispersed with the dispersant by sonication, and then were centrifuged to obtain a well-dispersed supernatant with large and un-exfoliated particles removed. Digital photographs of the dispersed solution before and after centrifugation are shown in Fig. 3a. The dispersions using no dispersant (only IPA) showed a moderate Tyndall effect, indicating that some exfoliation occurred. However, when the carboxylic acid and hydroxyl groups were used as the head groups of the dispersant, the Tyndall effect was reduced. The strongest Tyndall effect was found in the sample using the amine head group of the dispersant. The solubility and nano-dispersion of Nb_2_Se_9_ chains can be analysed using UV-vis-NIR spectroscopy (Fig. 3b). The Nb_2_Se_9_ with the amine head group showed the strongest absorption, indicating that a nano-dispersion of Nb_2_Se_9_ was obtained. The concentration of the dispersion in octadecylamine was measured using inductively coupled plasma (ICP) mass spectrometry to be 79.2 μg mL^−1^, which was about 1.6 times larger than for the sample without a dispersant (50.1 μg mL^−1^). When octadecylamine was used, the colloidal dispersion maintained for 7 days, which was slightly better than the 5 days maintaining without the dispersant **(**Fig. S1).

**Figure 4:**
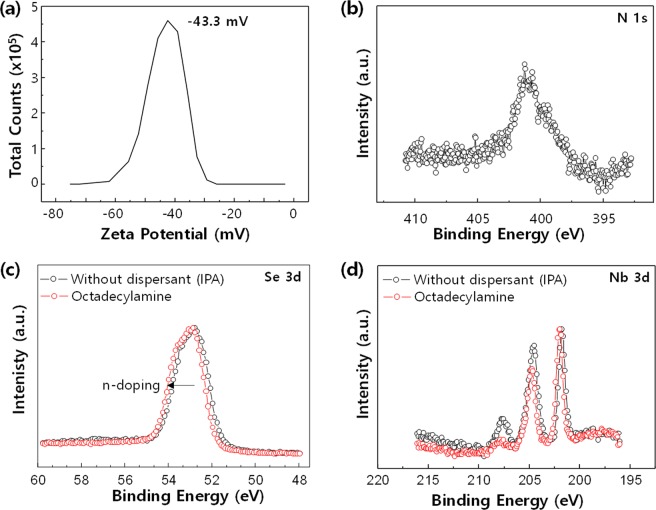
(**a**) Zeta potential of exfoliated Nb_2_Se_9_ in IPA. (**b**) XPS spectrum for Nb_2_Se_9_ with octadecylamine showing presence of N 1s peak. (**c**) XPS spectra of Se 3d peak without and with octadecylamine, showing upward shift of 0.5 eV in binding energy. (**d**) XPS spectra of Nb 3d peak without and with octadecylamine.

The adsorption of an organic polymer on inorganic materials in organic solvents can be explained through the acid-base theory (acidic polymers bind strongly to basic particles, while basic polymers bind strongly to acidic particles)^31–33^. Therefore, to understand the dispersion behaviour of Nb_2_Se_9_ crystals, it is necessary to understand the acid-base characteristics on the surface of Nb_2_Se_9_. The surface potential of Nb_2_Se_9_ in IPA was measured by a zeta potential meter and found to have a negative value of −43.3 mV (Fig. 4a), indicating that the surface of Nb_2_Se_9_ has acidic characteristics. Because Nb_2_Se_9_ has its own stable surface without dangling bonds, acid properties are induced by electrons localized on the surface of the material, not by various functional groups. Therefore, the acidic Nb_2_Se_9_ was better dispersed when the amine head group with a basic characteristic was used in the dispersant rather than the neutral hydroxyl or acidic carboxyl head group. This adsorption mechanism of the amine head group on the Nb_2_Se_9_ surface was confirmed by X-ray photoelectron spectroscopy (XPS) analysis. Figures 4b–d present the XPS core level spectra (N *1s*, Se *3d*, and Nb *3d*, respectively) to examine the charge transfer reaction between the amine head group of the dispersant and Nb_2_Se_9_. First, the N *1s* peak appeared clearly in the dispersed samples using octadecyl amine as a head. Second, a higher binding energy shift of about 0.5 eV occurred at the Se *3d* core level due to n-type doping when electrons were transferred from the amine head group to the electronegative Se atoms in the Nb_2_Se_9_ chains. When n-type doping occurs, the binding energy tends to increase because the Fermi energy level shifts to the conduction band and moves away from the core level. Since Nb atoms are surrounded by Se atoms in the Nb_2_Se_9_ chains, a higher binding energy shift also occurred at the Nb *3d* core level, but the XPS data were not clear enough to compare to Se *3d*. Based on these results, we conclude that the strong adsorption of the amine head group to Se atoms in the Nb_2_Se_9_ chains *via* charge transfer contributes to improving the dispersion of the Nb_2_Se_9_ chains.

**Figure 5:**
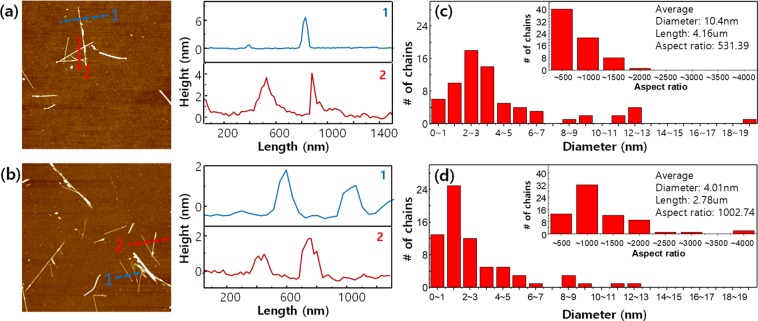
(**a,b**) AFM images of the exfoliated Nb_2_Se_9_ chains, and height profiles of dashed lines: (**a**) without dispersant, (**b**) with octadecylamine. (**c,d**) Histograms of the diameter distribution (inset: the aspect ratio) of Nb_2_Se_9_ solution deposited on Si/SiO_2_ wafer: (**c**) without dispersant, (**d**) with octadecylamine.

The dispersed Nb_2_Se_9_ was spin-coated on SiO_2_/Si substrates, and the sizes of the nano-chains were analysed using atomic force microscopy (AFM). The results are shown in Fig. 5a for pure IPA and Fig. 5b for octadecyl amine used as dispersant. These results confirm that mono-chains (~1 nm scale in AFM) of Nb_2_Se_9_ can be obtained by using an octadecyl amine dispersant instead of pure IPA. The effect of the dispersant can be more clearly seen in the statistical values of the AFM measurements (Fig. 5c,d). The average diameter of the Nb_2_Se_9_ chains in the pure IPA dispersion was about 10.4 nm, while that in the octadecyl amine dispersant was less than half that size, with a value of 4.0 nm. Also, the aspect ratio (length / diameter) of the chains when octadecyl amine dispersant was used was more than double that of the pure IPA dispersion (531.39 for pure IPA dispersion and 1002.74 for octadecyl amine dispersant).

## Conclusion

In summary, we designed the ideal dispersant to exfoliate Nb_2_Se_9_ inorganic molecular chains as 1D units from bulk crystals based on the acid-base theory. Zeta potential measurements confirmed that Nb_2_Se_9_ had acidic surfaces (negative zeta potential of about −43.3 mV). It was confirmed that octadecyl amine, with an amine head group with a basic property, bound more strongly to the Nb_2_Se_9_ surfaces than the acidic carboxyl or neutral hydroxyl head groups due to charge transfer between the amine and Se atoms, resulting in excellent dispersion to the nanometre level. The dispersant determined in this study is expected to be widely used for the study of new 1D Nb_2_Se_9_ materials.

## Methods

### Synthesis

Nb_2_Se_9_ was prepared by a flux method using elemental powders of Nb (325 mesh, 99.5%, Aldrich) and Se (99+ %, Alfa Aesar). A mixture of the starting elements in a molar ratio of Nb:Se = 1:200 was pelletised and then sealed in a 15 cm-long quartz tube with a neck in the middle of the tube. The evacuated quartz tube was heated to 800 °C for 72 h (at 5.5 °C h^−1^) and then naturally cooled to room temperature. Then, the unreacted Se flux was removed by inverting the quartz tube, causing the flux to fall to the other side of the tube, followed by heating in a box furnace at 250 °C for 12 h. Finally, the residual Se was sublimed in a tube furnace at 250 °C for 24 h under an Ar atmosphere. The resulting material was composed of grey needle-shaped crystals.

### Dispersion

First, 10 mg of the obtained Nb_2_Se_9_ was immersed in 20 mL of IPA. It was initially sonicated for 5 min with 2 s/2 s on/off intervals in a probe sonicator (VC 505, Sonics & Materials, Inc.) to crush the large crystals coarsely. After the first sonication, 10 mL of dispersant/IPA solution with a concentration of 1 mg mL^−1^ was added. Then, the solution containing the dispersant was sonicated again in a bath sonicator (B2005S-68K, 68 kHz, 200 W, KODO Technical) for 3 h. After the two ultrasonic steps, the solution was centrifuged at 6000 rpm for 10 min to remove the insufficiently dispersed chains. Finally, 10 mL of the supernatant solution was used for further analysis.

### Characterization

Field-emission scanning electron microscopy (FE-SEM, Hitachi, S-4300SE) was performed for morphological characterisation of Nb_2_Se_9_. Powder XRD (Mac Science, M18XHF22) was employed with Cu-K_α_ radiation (λ = 0.154 nm). A Zetasizer Nano-ZS90 from Malvern Instruments, Ltd. (Worcestershire, UK) and UV-vis spectrophotometer (Agilent Technologies lnc., Agilent 89090A) were used to measure the zeta potentials and UV absorption of the dispersions. X-ray photoelectron spectroscopy (XPS) data was obtained using an ESCALAB250 from Thermo. The samples were prepared by a filtering method using an Anodisc filter (Whatman) with a pore size of 100 nm to form a solid film. To evaluate the morphologies of the exfoliated nanowires, atomic force microscopy (AFM, Park systems, NX10) was employed in non-contact mode. The samples were prepared by spin-coating on SiO_2_/Si wafers.

## Supplementary information

Dispersion stability data

## Data Availability

The data that support the findings of this study is available from the corresponding author upon request.
