# Supplementary material for: Design of dispersant for highly concentrated one-dimensional Nb2Se9 inorganic molecular chains from bulk crystal
Source: Sci Rep. 2019 Oct 10;9:14579. doi: 10.1038/s41598-019-51210-z (PMC6787179; doi:10.1038/s41598-019-51210-z)
Supplement: Supplementary file 1 — Dispersion stability data [file 41598_2019_51210_MOESM1_ESM.docx]

**Design of dispersant for highly concentrated one-dimensional Nb_2_Se_9_ inorganic molecular chains from bulk crystal**

Sudong Chae^1,#^, Akhtar J. Siddiqa^1,#^, Seungbae Oh^1^, Bum Jun Kim^2^, Kyung Hwan Choi^2^, Hak Ki Yu^3,^*, Jae-Young Choi^1,2,^*

^1^School of Advanced Materials Science & Engineering, Sungkyunkwan University, Suwon, 16419, Korea

^2^SKKU Advanced Institute of Nanotechnology (SAINT), Sungkyunkwan University, Suwon, 16419, Korea.

^3^Department of Materials Science and Engineering & Department of Energy Systems Research, Ajou University, Suwon, 16499, Korea

^#^ These authors contributed equally to this work.

*Correspondence and requests for materials should be addressed to J.-Y.C. and H.K.Y.: [jy.choi@skku.edu](mailto:jy.choi@skku.edu) & [hakkiyu@ajou.ac.kr](mailto:hakkiyu@ajou.ac.kr)


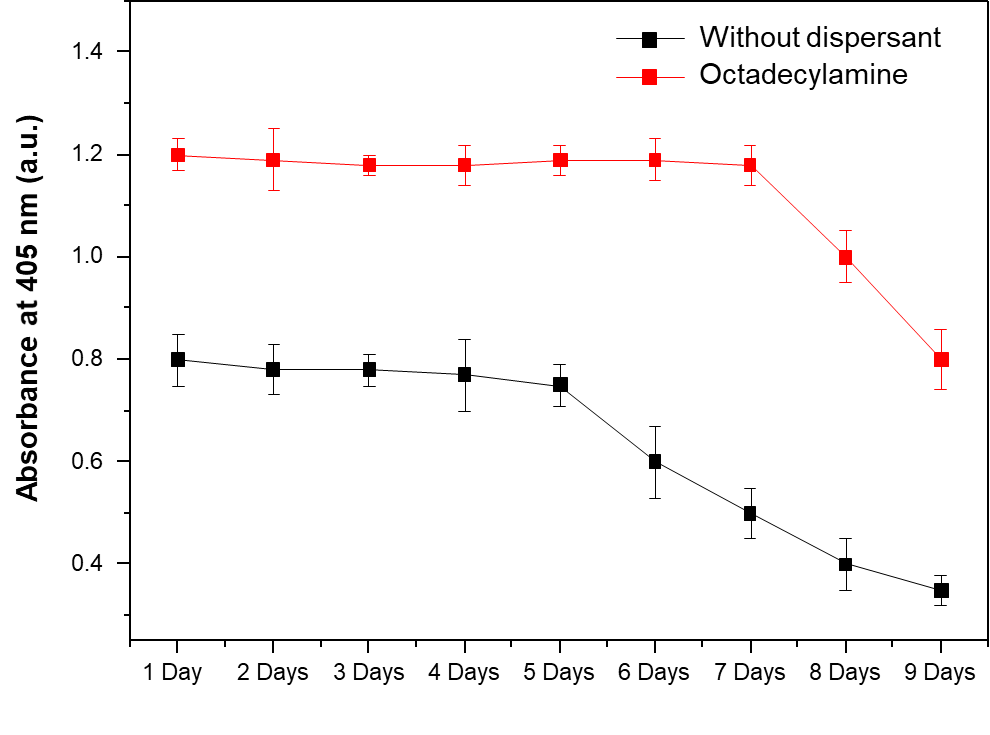
**Figure S1.** Absorbance at 405nm of the dispersion solutions.
